# Effect of terminal accuracy requirements on temporal gaze-hand coordination during fast discrete and reciprocal pointings

**DOI:** 10.1186/1743-0003-8-10

**Published:** 2011-02-14

**Authors:** Romain Terrier, Nicolas Forestier, Félix Berrigan, Mathieu Germain-Robitaille, Martin Lavallière, Normand Teasdale

**Affiliations:** 1 Laboratoire de Physiologie de l'Exercice (E.A. 4338), Département STAPS, UFR CISM, Université de Savoie, 73376 Le Bourget du lac cedex, France; 2 Groupe de Recherche en Analyse du Mouvement et Ergonomie, Division de Kinésiologie, Département de Médecine Sociale et Préventive, Faculté de Médecine, Université Laval, Québec (Québec), G1K 7P4, Canada; 3 Faculté d'éducation physique et sportive, Université de Sherbrooke, Sherbrooke (Québec), J1K 2R1, Canada

## Abstract

**Background:**

Rapid discrete goal-directed movements are characterized by a well known coordination pattern between the gaze and the hand displacements. The gaze always starts prior to the hand movement and reaches the target before hand velocity peak. Surprisingly, the effect of the target size on the temporal gaze-hand coordination has not been directly investigated. Moreover, goal-directed movements are often produced in a reciprocal rather than in a discrete manner. The objectives of this work were to assess the effect of the target size on temporal gaze-hand coordination during fast 1) discrete and 2) reciprocal pointings.

**Methods:**

Subjects performed fast discrete (experiment 1) and reciprocal (experiment 2) pointings with an amplitude of 50 cm and four target diameters (7.6, 3.8, 1.9 and 0.95 cm) leading to indexes of difficulty (ID = log_2_[2A/D]) of 3.7, 4.7, 5.7 and 6.7 bits. Gaze and hand displacements were synchronously recorded. Temporal gaze-hand coordination parameters were compared between experiments (discrete and reciprocal pointings) and IDs using analyses of variance (ANOVAs).

**Results:**

Data showed that the magnitude of the gaze-hand lead pattern was much higher for discrete than for reciprocal pointings. Moreover, while it was constant for discrete pointings, it decreased systematically with an increasing ID for reciprocal pointings because of the longer duration of gaze anchoring on target.

**Conclusion:**

Overall, the temporal gaze-hand coordination analysis revealed that even for high IDs, fast reciprocal pointings could not be considered as a concatenation of discrete units. Moreover, our data clearly illustrate the smooth adaptation of temporal gaze-hand coordination to terminal accuracy requirements during fast reciprocal pointings. It will be interesting for further researches to investigate if the methodology used in the experiment 2 allows assessing the effect of sensori-motor deficits on gaze-hand coordination.

## Background

The organization and control of goal-directed movements has been studied extensively using variations of the well known Fitts' task [1,2]. Within this general paradigm, the width of the target (W) and distance (A) of the movement are systematically varied across trials and subjects are asked to point at targets as rapidly and as accurately as possible. Generally, these studies have allowed to conclude that there is a linear relationship between the index of difficulty (ID = Log_2 _[2A/W]) and movement time (MT) (see [3,4] for reviews of this effect) with the MT increasing when the ID increases. It has been suggested the increase in MT corresponds to an increase of the amount of visual information that needs to be processed to generate a movement that would arrive at the target.

Rapid discrete goal-directed movements are characterized by a well known coordination pattern between the eye and the hand movement [5-7]. The gaze always starts prior to the hand movement and reaches the target at about the (i) hand movement onset [5,7], (ii) hand peak acceleration [8,9] or (iii) hand peak velocity [10-12]. Generally, the gaze is in the vicinity of the target during hand deceleration. Such a gaze-hand lead pattern is naturally assumed to allow (i) the early update of the initial hand motor plan on the basis of accurate target location encoding [13-15] and (ii) the control of the final phase of the movement on the basis of visual information about relative target and hand locations [9,16,17]. Surprisingly, the effect of the difficulty of the task (and hence of the target size) on the temporal gaze-hand coordination has not been directly investigated. It is certainly of interest (for instance, from a human factors perspective) to determine whether the reported gaze-hand organization, considered as optimal, is ID dependent.

Often, goal-directed movements are produced in a reciprocal rather than in a discrete manner. For instance, in the classical experiments of Paul Fitts, subjects pointed back and forth between two targets as fast and as accurately as possible for 20 sec. Despite the fact the linear relationship between the ID and movement time was first reported for reciprocal movements, there has been an ongoing debate about 1) whether the units of actions for discrete and reciprocal movements are similar [18-21], and 2) whether the relationship between the ID and movement time is linear [22]. For example, Guiard [19,23] showed that the deceleration phase of a reciprocal pointing completely overlaps the reacceleration phase of the following pointing movement, taking advantage of the stored elastic energy. Such a kinematic organization, governed by a cyclical unit, is qualified as harmonic (see [23] for details about harmonicity calculation) and Guiard [19] has argued this organization does not support the suggestion that reciprocal movements can be decomposed into discrete segments. This latter interpretation, often labeled the concatenation hypothesis, would imply a waste of this stored elastic energy once every half-cycle. Nevertheless, there are several examples where reciprocal pointings became inharmonic when the target size was decreased and the ID increased above a critical value included between 4.01 and 4.91 bits [23-25]. Recently, Huys et al. [22] also presented a demonstration that, for reciprocal movements, the relationship between ID and movement time is not continuous and that different control mechanisms correspond to low and high IDs with rhythmic movements implemented in easy tasks and discrete movements in difficult ones. This suggestion also has received support from neuro-imaging research [26,27]. For instance, Schaal et al. [26] reported that discrete wrist flexion and extension movements activated more cortical areas than rhythmic wrist movements. Specifically, more prefrontal and parietal areas were involved in reaching and complex sequential actions than for rhythmic movements, suggesting that rhythmic movements are monitored by an automatic control whereas more cognitive functions are required to control discrete movements.

As recently underlined by Lazzari et al. [28], the investigation of gaze-hand coordination during reciprocal tasks has received little attention despite the fact that for reciprocal movements, *visual information is required both to bring the movement in progress to a successful conclusion and to prepare the next movement *[29]. Hence, a trade-off has to be made between visual control of the final phase of the current movement and the magnitude of the gaze-hand lead pattern for the upcoming movement. Such a trade-off could potentially be influenced by the accuracy requirements (ID). According to Elliott et al. [30], when the accuracy requirements are relatively low, accurate movements may be concluded without visual information about relative target and hand locations during the terminal phase. Formally, larger targets could allow subjects to determine that the planned motor program (updated from accurate target location encoding) does not require terminal corrections. On the other hand, higher IDs would be associated with additional visual processing cost relative to the final phase of the preceding movement leading to a decrease of the gaze-hand lead pattern magnitude.

Two experiments were designed to analyze the effect of various IDs on the kinematics of the hand movement and the temporal coordination between the gaze and the hand. We examined the coordination of the gaze-hand lead pattern when fast discrete pointings and reciprocal pointings to four different target sizes were produced. Our results show a stable and fixed gaze-hand lead pattern for discrete pointings. For reciprocal pointings, the gaze-hand lead pattern was much smaller and decreased linearly with an increased target size. We discuss the role of this differential control mechanism for discrete and reciprocal movements.

## Experiment 1: discrete pointing

### Methods

#### Subjects

6 right handed males (mean age : 27 ± 3.8 yrs, mean height : 181 ± 5.5 cm, and mean weight: 77 ± 9.2 kg) without any history of joint or neuromuscular disease took part in this experiment on a voluntary basis. They were naïve as to the purposes of the experiment. All participants gave their written informed consent to participate in this study, which was approved by the Laval University Ethic Committee.

#### Task and apparatus

**Figure 1:**
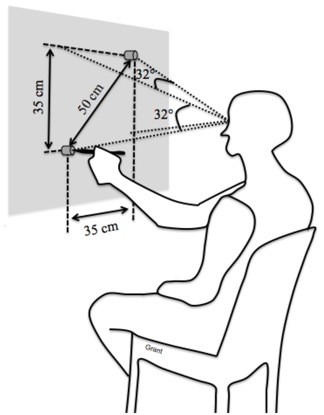
**Schematic of the experimental set up**. See the text for more details.

As illustrated in figure 1, participants were seated in front of a vertical board with two aluminum circular targets. The distance between subjects' forehead and the board was approximately 60 cm. The center of the lower target (T1) was about at the height corresponding to the subjects' inter-acromial line. The upper target (T2) was shifted 35 cm to the right and to the top leading to amplitude (A) of 50 cm between targets. Thus, the horizontal and vertical amplitudes of gaze displacements necessary to focus on each target's center were about 32°. A Fitts-like paradigm (Fitts 1954) with four pairs of targets (diameter (D) of 7.6, 3.8, 1.9 and 0.95 cm; thickness : 2.5 cm) was used for the pointing trials. This setup allowed indices of difficulty (ID = log_2_[2A/D]) of 3.7, 4.7, 5.7 and 6.7 bits. Pointing movements were made with a stylus having a 1-mm tip. The targets and the stylus were electrically connected allowing detection of when subjects left the lower target and reached the upper one. This voltage signal was recorded at 1200 Hz (12-bit A/D conversion). Moreover, the 3D kinematics of the effectors movement was sampled at 120 Hz by means of a magnetic receiver (Polhemus™ Liberty) fixed on the stylus.

The eye and head movements were recorded with a head mounted eye tracker (Applied Sciences Laboratories model H6). The eye camera and infra-red illuminator enabled tracking the left eye pupil and corneal reflection with a real-time delay of 25 ms. A calibration procedure specific to each subject allowed determining the eye-in-head position within a 45° (horizontal) by 35° (vertical) visual field. A magnetic receiver (Flock of Birds Ascension Technology) fixed on the eye tracker headband recorded the head position and orientation in space. Finally, the eye tracker system integrated the eye-in-head and head-in-space positions, both sampled at 120 Hz, to compute the point of gaze coordinates on the vertical board plane.

All data (target contacts, kinematics of the stylus, and point of gaze coordinates) were synchronized on the external sync TTL signal of the Polhemus Liberty by means of a microcontroller (Parallax, Basic Stamp).

#### Procedure

For each of the four IDs, subjects performed a block of ten discrete pointing trials from the lower (T1) to the upper (T2) target. The order of presentation was randomized between subjects. They were instructed to point as quickly and accurately as possible. Each trial started with the stylus and the point of gaze on the lower target. A verbal signal given by the experimenter was the stimulus to move. A trial was accepted when the subject hit the target without any contact with the surrounding board. The targets' thickness (2.5 cm) precluded subjects from gliding between the vertical board and the stylus. Subjects were not allowed more than 2 errors per block. When this occurred, a new condition was presented and the complete block of 10 trials was presented again at the end of the session. To prevent fatigue, a short rest was allowed between each trial and each block. Before data recording, subjects performed several discrete pointing trials until they feel comfortable and efficient for the different IDs.

#### Data analysis

The electrical contacts between the stylus and the targets were used to determine the start and the end of each pointing trial. The duration between the end of the lower target contact and the onset of the upper target contact was defined as the hand movement time (MT).

Position data from the stylus were filtered (Butterworth fourth-order with a 7 Hz low pass cut-off frequency with dual-pass to remove phase shift) prior to calculation of the hand resultant velocity (finite-difference algorithm). Velocity peaks were determined with custom software developed in Matlab™. The duration between the onset of a pointing and its peak speed defined the duration of the acceleration phase while the time between the peak speed and the end of the pointing defined the duration of the deceleration phase.

The onset of gaze displacement for each pointing was determined from the resultant velocity in the vertical plane using a threshold of 1 m.s^-1 ^[31]. The ONSET latency, defined as the difference between the onset of the gaze displacement and that of the hand was then calculated as follows:

ONSET latency=onset of the hand−onset of the gaze.

A positive value indicates the gaze displacement was initiated prior to the hand movement whereas a negative value indicates the gaze was initiated after the hand movement.

All dependent variables were submitted to one-way repeated measures ANOVA (4 IDs). A .05 alpha threshold was adopted throughout. When significant, the main effect of ID was decomposed with a linear trend analysis.

### Results

#### Hand movements characteristics

Table 1 presents a summary of the results for the discrete pointings. Overall, we recorded 14 errors and only 2 blocks were retaken. Movement time, the duration of acceleration and deceleration phases all increased with an increasing ID while hand peak speed decreased. Post-hoc analyses showed that the increase was linear for MT, and the deceleration phase duration; the decrease was linear for the hand peak speed (linear trends analyses: F(1,5) = 126.6, *p *< 0.01; F(1,5) = 99.3, *p *< 0.01; F(1,5) = 25.45, *p *< 0.01, respectively). For the acceleration phase duration, the linear trend was not significant (F(1,5) = 4.75, *p *> 0.05) but the durations for the two smaller IDs were smaller than those for the two larger IDs (*ps *< 0.05) The deceleration phase duration expressed in percentage of the movement time increased significantly with an increasing ID, illustrating that hand movements became less symmetric when the ID increased. On average, for the lower and higher ID, the deceleration phase duration represented 59% and 79% of the movement time, respectively.

**Table 1:** Effects of ID on temporal parameters of hand movements during discrete pointing.

Temporal parameters	ID values				Effect of ID	
	**3.7**	**4.7**	**5.7**	**6.7**	**F(3,15)**	
**Movement time (ms)**	276 (±36)	335 (±36)	512 (±47)	651 (±69)	92.9	***
**Duration of acceleration phase (ms)**	113 (±17)	114 (±8)	133 (±12)	134 (±16)	4.4	*
**Duration of deceleration phase (ms)**	163 (±33)	221 (±31)	379 (±48)	517 (±72)	74.8	***
**Duration of deceleration phase (%MT)**	59 (±6)	65.5 (±2.5)	74 (±3)	79 (±3.5)	26.8	***
**Hand velocity peak (m.s**^**-1**^**)**	2.93 (±0.34)	2.60 (±0.14)	2.30 (±0.12)	2.19 (±0.11)	16.7	***

*** *P *< 0.001; * *P *< 0.05

#### Gaze-hand coordination

All ONSET latencies were positive indicating that gaze displacement was initiated systematically prior to the hand movement. The main effect of ID was not significant (F(3,15) = 0.12, *p *= 0.95) and the mean ONSET latency was 145 ms.

### Discussion

As stipulated by Fitts' law, MT for discrete pointings increased linearly with an increasing ID. A more detailed analysis of the hand responses (see Table 1) revealed that the increased MT resulted mostly from an increased duration of the deceleration phase. As reported by several authors (e.g. [3,32]), this presumably results from an increased reliance upon visual feedback control processes for the most difficult IDs.

**Figure 2:**
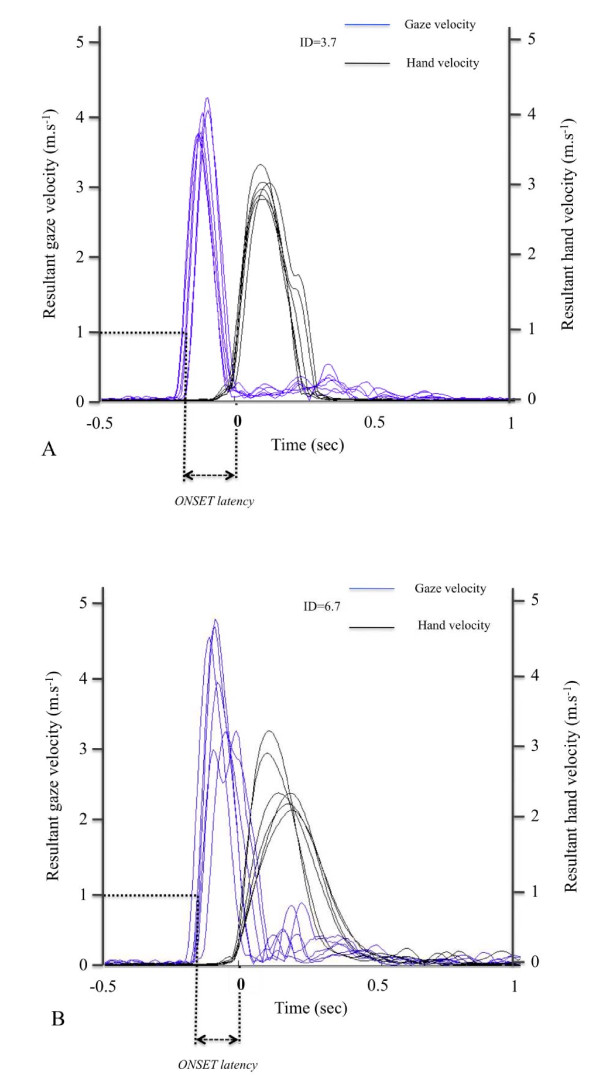
**Typical data of one representative subject for discrete pointing trials**. (**A**) 3.7 bits ID condition. (**B**) 6.7 bits ID condition. Blue lines represent gaze velocity profiles whereas black lines represent hand velocity profiles. Note that ONSET latency was stable across ID conditions and that gaze was anchored on target before hand velocity peak.

Varying the size of the target did not modify the ONSET latency and the gaze was initiated, on average, 145 ms prior to the onset of the hand movement. This confirms previous observations with various aiming and pointing tasks (e.g.[5,7-9,33]). Figure 2 shows gaze and hand velocity profiles from one representative subject, for the lower (2A) and the higher (2B) IDs. These data illustrate that ONSET latency was stable and that gaze was anchored on the target before the hand peak velocity. As mentioned above, this sequence allows both (i) the early update of the initial hand motor plan on the basis of an accurate encoding of the target location [13-15] and (ii) an accurate control of the final phase of the pointing movement on the basis of visual information about relative target and hand locations [9,16].

The second experiment examines if this fixed organization remains when reciprocal pointings are performed. As mentioned in the introduction there has been an ongoing debate as to whether the units of actions for discrete and reciprocal movements are similar [18-21,23]. If reciprocal pointings for higher IDs are a succession of real discrete units, a similar and stable gaze-hand lead pattern should be observed even when pointing to smaller targets and this gaze-hand pattern should resembled that observed for discrete movements. If this is the case, an increased visual processing relative to the final phase of the preceding movement could be associated with a gaze-hand lead magnitude stabilization by means of a dwell time increase [24,34,35].

## Experiment 2: reciprocal pointings

### Methods

#### Subjects

12 right handed males (mean age: 25.2 ± 4.7 yrs, mean height: 179.6 ± 6.5 cm, and mean weight: 75.6 ± 8.2 kg) took part in this study. Six of them also participated in experiment 1. As for experiment 1, they were without any history of joint or neuromuscular disease and they took part in the experiment on a voluntary basis. They were all naïve as to the specific purposes of the experiment. All participants gave their written informed consent to participate in this study, which was approved by the Laval University Ethics Committee.

#### Task and apparatus

The same experimental set-up was used and the two studies were differentiated only by the nature of the pointing task: discrete pointings in experiment 1 and reciprocal pointings in this second experiment.

#### Procedure

For each ID, the task was to alternatively point at the targets as quickly and as accurately as possible during a 25 seconds trial. As the error level cannot easily be controlled online during reciprocal pointings, a ratio of unsuccessful/successful contacts was calculated a posteriori. No more instruction was given in order to record the subjects' visuo-motor organizations under unconstrained conditions. Before data recording, subjects performed practice trials until they felt comfortable and efficient for the different IDs. During data recording, the order of presentation of the four targets (IDs) was randomized between subjects. Each trial started with the stylus and the point of gaze on the lower target. To prevent fatigue, a short rest was allowed between trials and target conditions.

#### Data analysis

As for experiment 1, the contact signals and hand displacement data were processed to compute Movement Time (MT), hand peak velocity, and duration of the acceleration and deceleration phases. All trials were visually inspected by comparing contact signals to hand displacement signals. When a hand reversal displacement (as observed from the displacement signals from the magnetic tracker) was not associated with a target contact, the pointing was considered as unsuccessful. To determine pointing accuracy, the ratio of unsuccessful pointings (without target contact) to the total number of pointings was calculated for each 25-s trial. Moreover, the contact time (CT), defined as the time between the onset and the end of the same target contact, was also computed.

The temporal gaze-hand coordination was analyzed by computing the ONSET latency with the same methodology than for the first experiment. To avoid the analysis of initial responses starting from a static position and the last responses where subjects may have anticipated the end of the 25-s period, the gaze responses for the first ten successful pointings (with target contact) between the 7th and 18th second were analysed. Moreover, a supplementary variable (OFFSET latency) specific to reciprocal pointings also was computed. The OFFSET latency was defined as the difference between the end of the hand movement (n) and the onset of the gaze for the following movement (n + 1). It was calculated as follows:

OFFSET latency=end of hand movement (n)–onset of gaze (n+1)

**Figure 3:**
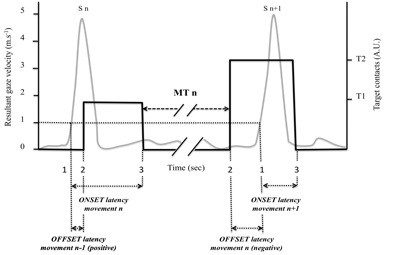
**Illustration of the methodological approach to compute ONSET and OFFSET latencies**. The black line represents the contacts between the stylus and the targets. The grey line represents resultant gaze velocity in the vertical plane. Numerical marks are defined as follows: 1 = onset of gaze saccade; 2 = end of the preceding hand movement; 3 = onset of the considered hand movement. Note that OFFSET latency of the movement n-1 is positive (saccade n began before the end of movement n-1) whereas the OFFSET latency of the movement n is negative (saccade n + 1 began after the end of movement n). It can also be observed that ONSET latency for movement n is longer than ONSET latency for movement n + 1.

A positive value indicates the gaze moved on to the next target before completion of the preceding pointing whereas a negative value indicates the gaze still focused on the currently aimed target when the hand made contact with the target. Figure 3 illustrates how ONSET and OFFSET latencies were computed.

All dependent variables were submitted to one-way repeated measures ANOVA (4 IDs). Furthermore, for the 6 subjects who performed the two experiments, a specific 2 Conditions (discrete and reciprocal pointings) × 4 IDs (3.7, 4.7, 5.7 and 6.7 bits) ANOVA with repeated-measures on both factors was performed on ONSET gaze-hand latency. A .05 alpha threshold was adopted throughout. When significant, the main effect of ID was decomposed with a linear trend analysis.

### Results

#### Hand movement characteristics

The percentage of unsuccessful pointings increased significantly with an increasing ID but values remained relatively low (on average, 1.9, 3.0, 7.1 and 7.0% for IDs of 3.7, 4.7, 5.7 and 6.7 bits, respectively; F(3,33) = 4.18, *p *< 0.01). A comparison of means (Tukey) showed the percentages were not different for the two lower IDs (*p *> 0.05) and that percentages for the two higher IDs were greater than those observed for the smaller IDs (*p *< 0.05). Table 2 presents a summary of the results for the pointings analyzed. As for discrete pointings, the main effect of ID was significant for all variables analysed. MT increased linearly with an increasing ID (F(1,11) = 159.1, *p *< 0.01 for the linear trend). Both the duration of the acceleration and deceleration phases also increased linearly with an increasing ID (F(1,11) = 144.4, *p *< 0.01 and F(1,11) = 207.2, *p *< 0.01, respectively) and the hand peak speed decreased linearly with an increasing ID (F(1,11) = 68.2, *p *< 0.01). Moreover, the deceleration phase duration expressed in percentage of the movement time increased significantly with an increasing ID, illustrating that hand movements became less symmetric. On average, the deceleration phase duration represented 54% and 67% of the movement time, for the lower and higher ID respectively. Finally, the duration of the contact with the targets (or dwell time) increased linearly with an increasing ID (F(1,11) = 50.8, *p *< 0.01). However, this increase of 27 ms from the lower to the higher ID was small.

**Table 2:** Effects of ID on temporal parameters of hand movements during reciprocal pointing.

Temporal parameters	ID values				Effect of ID	
	**3.7**	**4.7**	**5.7**	**6.7**	**F(3,33)**	
**Movement time (ms)**	299 (±50)	374 (±52)	508 (±69)	701 (±120)	118.2	***
**Duration of acceleration phase (ms)**	136 (±30)	157 (±27)	200 (±27)	231 (±31)	106.6	***
**Duration of deceleration phase (ms)**	163 (±31)	217 (±39)	308 (±49)	470 (±90)	133	***
**Duration of deceleration phase (%MT)**	54 (±5.5)	58 (±5)	61 (±4)	67 (±5.5)	43.5	***
**Hand velocity peak (m.s**^**-1**^**)**	2.55 (±0.24)	2.23 (±0.25)	1.95 (±0.20)	1.86 (±0.27)	35.8	***
**Contact time (ms)**	50 (±12)	53 (±8)	66 (±15)	77 (±19)	22.5	***

*** *P *< 0.001

#### Temporal gaze-hand coordination

##### ONSET latency during reciprocal pointings (12 subjects)

**Figure 4:**
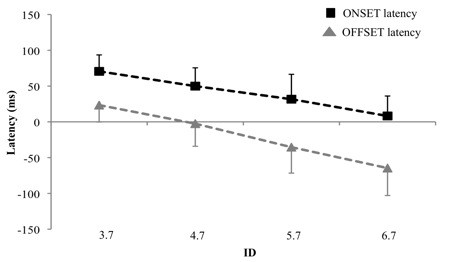
**Illustration of the effect of ID on ONSET and OFFSET latencies for reciprocal pointing trials**. Black squares represent ONSET latency whereas grey triangles represent OFFSET latency for the 12 subjects who performed the experiment 2. Error bars represent the standard deviation. Note that ONSET and OFFSET latencies significantly decreased with an increasing ID.

**Figure 5:**
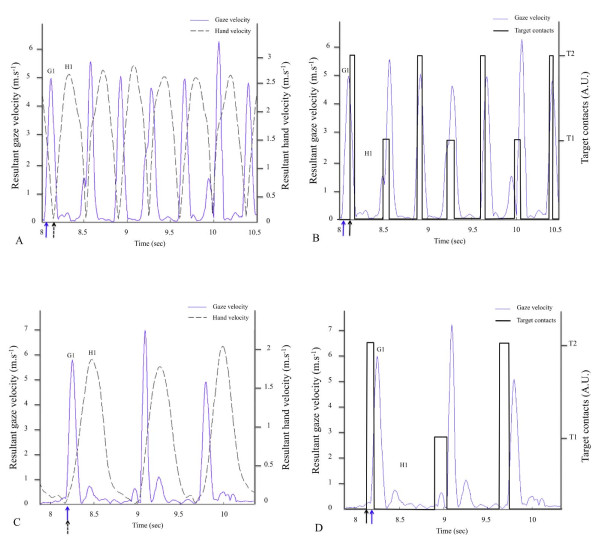
**Typical data of one representative subject for reciprocal pointing trials**. **A and B: lower ID (3.7 bits)**. Figure 5A presents gaze (blue solid line) and hand (black dashed line) velocity profiles. The blue solid arrow represents gaze onset time and the black dashed arrow represents hand onset time for the same pointing. Figure 5B presents gaze velocity and targets contacts (dark square-like signals) for the same pointings. The blue solid arrow represents gaze onset time and the black solid arrow represents the end of the preceding hand movement. **C and D: higher ID (6.7 bits)**. Figure 5C presents gaze (blue solid line) and hand (black dashed line) velocity profiles. The blue solid arrow represents gaze onset time and the black dashed arrow represents hand onset time for the same pointing. Figure 5D presents gaze velocity and targets contacts (dark square-like signals) for the same pointings. The blue solid arrow represents gaze onset time and the black solid arrow represents the end of the preceding hand movement. See the text for more details.

Figure 4 presents the average ONSET latency for the four IDs during the reciprocal pointing. All ONSET latencies were positive indicating that gaze displacements were systematically initiated prior to hand movements. The ANOVA revealed a significant effect of ID (F(3,33) = 42.64, *p *< 0.01) and, as illustrated in figure 4, the mean ONSET latency decreased linearly with an increasing ID (F(1,11) = 114.8, *p *< 0.01 for the linear trend). This also indicates the magnitude of gaze-hand lead pattern was reduced when the difficulty of the task (ID) increased and it was nearly abolished for the most difficult ID. A t-test showed the ONSET latencies for the 6.7 bits ID were not different from 0 (t(11) = 1.02, *p *> 0.05) suggesting the gaze and hand were nearly synchronous. This modification of the temporal gaze-hand coordination is illustrated in figures 5A and 5C. Figure 5A presents gaze (blue solid line) and hand (black dashed line) velocity profiles for 6 pointings for the lower ID (3.7 bits) condition. Gaze onset times precede hand onset times, corresponding to positive ONSET latencies. For example, the first gaze onset time (G1, blue solid arrow) precedes the first hand onset time (H1, black dashed arrow). Figure 5C presents gaze (blue solid line) and hand (black dashed line) velocity profiles for 3 pointings for the higher ID (6.7 bits) condition. Gaze and hand onset times are nearly synchronous. For example, the first gaze onset time (G1, blue solid arrow) occurs only few milliseconds before the first hand onset time (H1, black dashed arrow).

##### OFFSET latency during reciprocal pointings (12 subjects)

As shown in figure 4, the OFFSET latency also decreased with an increasing ID. The ANOVA showed a significant effect of ID on OFFSET latency (F(3,33) = 51, *p *< 0.01) and the linear trend was significant (F(1,11) = 154.5, *p *< 0.01). The OFFSET latencies were positive for the ID of 3.7, indicating that subjects moved their gaze on to the next target before completing the preceding movement. However, for higher IDs, subjects still fixated the aimed target at the contact time, as revealed by the negative values of OFFSET latency. As the ID increased, subjects increased the duration of the fixation on the target. This differential gaze-hand organization is well illustrated by representative data displayed on figures 5B and 5D showing gaze velocity profiles (blue solid lines) and targets contacts (dark square-like signals). The beginning of a target contact corresponds to the end of a hand movement whereas the end of a target contact represents the beginning of the following hand movement. Figure 5B shows positive OFFSET latencies associated with the smaller ID: for most pointings, the gaze displacement begins before the end of the preceding hand movement. For example, the onset time of the first gaze displacement (G1, blue solid arrow) occurs before the end of the preceding hand movement (black solid arrow). Figure 5D shows negative OFFSET latencies associated with the higher ID: gaze displacements usually begin after the end of the preceding hand movement. For example, the first gaze displacement (G1, blue solid arrow) begins after the end of the preceding hand movement (black solid arrow). This is observed for all three gaze responses illustrated.

#### Discrete vs reciprocal pointings (6 subjects)

**Figure 6:**
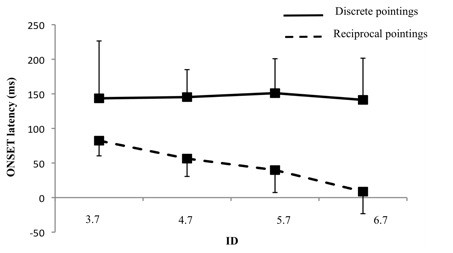
**Illustration of the effect of ID on ONSET latencies for discrete and reciprocal pointings**. Data from the 6 subjects who performed the two experiments, i.e. discrete and reciprocal pointings are presented. The solid line represents discrete pointing whereas the dashed line represents reciprocal pointing. Error bars represent the standard deviation. Note (i) that ID affects ONSET latency for reciprocal but not for discrete pointing (ii) and that values are significantly higher for discrete than for reciprocal pointing for all IDs.

The ONSET latencies for the 6 subjects who participated to both experiments (discrete and reciprocal pointings) were compared to directly assess differences in temporal gaze-hand coordination between discrete and reciprocal pointings. As illustrated in figure 6, the comparison of ONSET latencies obtained during experiment 1 (discrete pointing task) and 2 (reciprocal pointing task) revealed a significant main effect of Task (F(1,5) = 19.23, *p *< 0.01) showing that ONSET latencies were globally higher for the discrete pointing task than for the reciprocal task. The ANOVA also showed a significant interaction of Task × ID (F(3,15) = 5.35, *p *< 0.05) illustrating that, while the gaze-hand lead pattern was constant for all IDs for discrete pointings, the ONSET latency was smaller for reciprocal pointings and it decreased with an increasing ID. The ONSET latency was almost zero for the 6.7 bits conditions. These changes in the gaze-hand coordination suggest that, from a visuo-manual viewpoint, fast reciprocal pointings under high IDs conditions could not be considered as a concatenation of discrete units.

## Discussion

The a posteriori analysis of the errors showed a significant effect of ID on the ratio of unsuccessful trials. This ratio was small (less than 2%) when accuracy constraints were smaller (3.7 bits) and it increased somewhat (up to 7%) when accuracy constraints increased (6.7 bits). Despite this small decrease in the accuracy, as stipulated by Fitts' law, MT still increased linearly as a function of the increasing ID suggesting that subjects respected both the speed and the accuracy instructions. The increased MT resulted mostly but not exclusively from an increased duration of the deceleration phase. Expressed in percentage of movement time, this increase shows that hand movement kinematics became less symmetric with an increasing ID. In addition, a small but significant increase of dwell times was observed with an increasing ID (on average, 27 ms from the lower to the higher ID).

This small increase in dwell time did not lead to constant and stable gaze-hand coordination. With increasing ID, significant and gradual changes were observed in the gaze-hand coordination. From a visuo-manual viewpoint, none of the patterns resembled that observed for discrete movements suggesting that reciprocal pointings were not a concatenation of discrete units at any of the ID examined. Specifically, the temporal analysis of the gaze-hand coordination revealed that the OFFSET latency decreased when the ID increased. The mean OFFSET latency was small but positive for the 3.7 bits ID (about 20 ms) whereas it was negative for the 6.7 bits ID (about -60 ms). Hence, this indicates the gaze moved to the next target before completion of the preceding movement when the ID was small whereas subjects still focused on the aimed target at the contact time for higher IDs. The positive OFFSET latency associated with the smaller ID is consistent with previous observations showing that visual information about the final phase is not necessary to conclude movements towards large targets [30,36,37]. The negative OFFSET latencies associated with higher IDs suggest that visual feedback from both hand and target during movement termination was necessary for coping with the increased accuracy requirements. Such a result is in line with the data reported by Neggers and Bekkering [6,38] for discrete movements showing that gaze often is anchored on the aimed target until movement completion. This was also observed in experiment 1, as illustrated in figure 2. Neggers and Bekkering [6] showed that anchoring was present even without vision of the moving limb suggesting the mechanism is based either on an internal signal or a proprioceptive signal. The small increase of contact time from lower to higher ID (on average, 27 ms) was not sufficient to compensate for the increasing visually-based control of the final movement stage and planning of the upcoming movement. As a result, the ONSET latency decreased significantly with an increasing ID indicating that the magnitude of the gaze-hand lead pattern was reduced when the target size decreased. On average, the gaze onset preceded the hand onset by about 70 ms for the 3.7 bits ID whereas gaze and hand onsets occurred nearly simultaneously for the 6.7 bits ID.

Interestingly, this gaze and hand onset times synchronization is not in agreement with the classical gaze-hand lead pattern reported for discrete pointings [5,8-11] which was also observed in experiment 1 with discrete pointings. Data from the 6 subjects who participated to both experiments allowed a direct comparison of ONSET latencies for discrete and reciprocal pointings. First, the magnitude of the gaze-hand lead pattern was much higher for discrete than for reciprocal pointings. Second, while it was constant for discrete pointings, it decreased systematically with an increasing ID for reciprocal pointings (nearly simultaneous onset for the gaze and hand for the 6.7 bits ID). These observations, at least from a visuo-manual viewpoint, clearly suggest that rapid reciprocal pointings cannot be considered as a succession of discrete movements. This also agrees with recent observations by Huys et al. [22] about reciprocal movements. These authors have argued that Fitts' law is discontinuous in reciprocal aiming. They suggested a transition in the control mechanisms with increasing task difficulty, with rhythmic movements implemented in easy tasks and discrete movements in difficult ones. Our data also favors this interpretation. There is a possibility that for lower IDs, an intermittent mode of control was adopted. Such a mode certainly is reminiscent of the occasional executive monitoring proposed by Pew [39] more than four decades ago. As the task difficulty increases, an increased visual control over the terminal phase of the movement is observed. It is this increased reliance upon visual control that leads to a transition in the control mechanism. The discontinuity observed as the task difficulty increases, however, is not a concatenation of discrete units observed when discrete movements only are planned and controlled. The dual role of vision in reciprocal movements (control of the terminal phase and planning of the upcoming movement) appears to impose additional constraints that are not implemented simply by increasing the dwell time. The more rapid gaze response still allows the gaze to be in the vicinity of the target as the hand approaches it. Lazzari et al. [28] recently observed that eye-hand synchronization switched from intermittent control for larger targets to continuous monitoring for small targets. The temporal analysis reported here shows a more progressive adaptation of the gaze-hand coordination as a function of the endpoint accuracy requirements.

## Conclusion

The aim of this work was to determine whether accuracy constraints altered the gaze-hand coordination pattern when producing discrete or reciprocal pointings. For discrete pointings, a robust and stable temporal visuo-manual coordination was observed with the gaze leading the hand by about 145 ms. When performing fast reciprocal pointings, the duration of the gaze anchoring on the target increased by approximately 85 ms from the lower to the higher ID and the contact duration (or dwell time) increased, on average by only 27 ms. As a consequence, the magnitude of the gaze-hand lead pattern decreased and it was nearly abolished for the higher ID (6.7 bits). Overall, the temporal gaze-hand coordination analysis revealed that even for high IDs, fast reciprocal pointings could not be considered as a concatenation of discrete units.

Finally, our data clearly illustrate the smooth adaptation of temporal gaze-hand coordination to contextual parameters such as terminal accuracy requirements during fast reciprocal pointings. It will be interesting for further researches to investigate if the methodology used in the experiment 2 allows assessing the effect of sensori-motor deficits on gaze-hand coordination. In the future, such a procedure may be used to accurately assess visuo-motor deficits in patients suffering from pathologies such as Parkinson's disease [40], cerebral palsy [41] or traumatic brain injuries [42], known to impair visuo-motor coordination.

## Competing interests

The authors declare that they have no competing interests.

## Authors' contributions

RT, NF and NT conceived the study. RT recruited subjects, performed data acquisition and analysis and drafting of the manuscript. NF and NT evaluated the data and participated to the manuscript writing. FB, MGR and ML participated to data acquisition and analysis. All authors read and approved the final manuscript.
